# Internalization of Modified Lipids by CD36 and SR-A Leads to Hepatic Inflammation and Lysosomal Cholesterol Storage in Kupffer Cells

**DOI:** 10.1371/journal.pone.0034378

**Published:** 2012-03-28

**Authors:** Veerle Bieghs, Fons Verheyen, Patrick J. van Gorp, Tim Hendrikx, Kristiaan Wouters, Dieter Lütjohann, Marion J. J. Gijbels, Maria Febbraio, Christoph J. Binder, Marten H. Hofker, Ronit Shiri-Sverdlov

**Affiliations:** 1 Department of Molecular Genetics, Electron Microscopy Unit of Molecular Cell Biology, and Pathology, Nutrition and Toxicology Research (NUTRIM) and Cardiovascular Research (CARIM) Institutes of Maastricht, University of Maastricht, Maastricht, The Netherlands; 2 Inserm U1011, UDSL, Institut Pasteur de Lille, University Lille Nord de France, Lille, France; 3 Institute of Clinical Chemistry and Clinical Pharmacology, University of Bonn, Bonn, Germany; 4 Department of Molecular Cardiology, Lerner Research Institute, Cleveland, Ohio, United States of America; 5 Center for Molecular Medicine, Austrian Academy of Sciences, and Department of Laboratory Medicine, Medical University of Vienna, Vienna, Austria; 6 Department of Pathology & Laboratory Medicine, University Medical Center Groningen, University of Groningen, Groningen, The Netherlands; Massachusetts General Hospital and Harvard Medical School, United States of America

## Abstract

**Background & Aims:**

Non-alcoholic steatohepatitis (NASH) is characterized by steatosis and inflammation, which can further progress into fibrosis and cirrhosis. Recently, we demonstrated that combined deletion of the two main scavenger receptors, CD36 and macrophage scavenger receptor 1 (MSR1), which are important for modified cholesterol-rich lipoprotein uptake, reduced NASH. The individual contributions of these receptors to NASH and the intracellular mechanisms by which they contribute to inflammation have not been established. We hypothesize that CD36 and MSR1 contribute independently to the onset of inflammation in NASH, by affecting intracellular cholesterol distribution inside Kupffer cells (KCs).

**Methods & Results:**

*Ldlr^−/−^* mice were transplanted with wild-type (Wt), *Cd36^−/−^* or *Msr1^−/−^* bone marrow and fed a Western diet for 3months. *Cd36^−/−^*- and *Msr1^−/−^*- transplanted (tp) mice showed a similar reduction in hepatic inflammation compared to Wt-tp mice. While the total amount of cholesterol inside KCs was similar in all groups, KCs of *Cd36^−/−^*- and *Msr1^−/−^*-tp mice showed increased cytoplasmic cholesterol accumulation, while Wt-tp mice showed increased lysosomal cholesterol accumulation.

**Conclusion:**

CD36 and MSR1 contribute similarly and independently to the progression of inflammation in NASH. One possible explanation for the inflammatory response related to expression of these receptors could be abnormal cholesterol trafficking in KCs. These data provide a new basis for prevention and treatment of NASH.

## Introduction

Non-alcoholic steatohepatitis (NASH) is increasingly recognized as a major health burden in developed countries, with a prevalence of 2–3% in the general population and up to 37% of the severely obese population of Western countries [1]. It is characterized by hepatic lipid accumulation (steatosis) combined with inflammation. While steatosis itself is generally considered benign, the presence of inflammation can lead to progression of NASH, resulting in liver fibrosis, cirrhosis and eventually liver failure and hepatocellular carcinoma [2]. The inflammatory response in the liver represents a key step in NASH development as it contributes to further liver damage by cirrhosis and hepatocellular carcinoma.

We have previously shown that hyperlipidemic mice fed a Western diet show an early onset of hepatic inflammation, which is associated with bloated Kupffer cells (KCs). These KCs resemble the foam cells of atherosclerotic lesions [3]. The current view favours a model where scavenger receptors (SR) mediate uptake of oxidized lipoproteins by macrophages, leading to foam cell formation and initiation of the inflammatory response. In line with this, we have already shown that the haematopoietic deletion of scavenger receptors *Cd36* and macrophage scavenger receptor 1 (*Msr1*) in low density lipoprotein (LDL) receptor-deficient (*Ldlr^−/−^*) mice attenuates hepatic inflammation [4]. However, while hepatic inflammation, lipid oxidation and liver fibrosis were significantly reduced, these mice showed no difference in foamy KCs. Similarly, it has also been shown that deletion of CD36, MSR1 or both do not completely abrogate foam cell formation *in vitro* or *in vivo*. In terms of atherosclerosis, there is controversy as to whether there are reduced lesions in *apoE/Cd36/Msr1* triple knock-out mice, but there is consensus that absence of these receptors reduced lesion complexity and inflammation [5], [6]. Thus, in contrast to the current view, these observations suggest that scavenger receptors modulate inflammation without altering total lipid accumulation in macrophages. Likewise, there is a growing appreciation of the numerous similar but independent functions of each one of these scavenger receptors in addition to lipoprotein uptake, including their role in inflammatory signal transduction and tissue homeostasis [7]. Currently, the individual contributions of these receptors to the onset of inflammation and the intracellular mechanisms by which they contribute to inflammation have not yet been established. Based on their different functions in inflammatory signal transduction, we hypothesized that CD36 and MSR1 contribute independently to the onset of hepatic inflammation, by affecting intracellular cholesterol distribution inside KCs.

Under normal conditions, lipoproteins which are endocytosed by KCs will initially be directed to the lysosomes, where they are hydrolyzed by lysosomal enzymes and transferred into the cytoplasm. However, the uptake of oxLDL by macrophages, which is modulated by CD36 and MSR1, is associated with lysosomal trapping *in vitro* [8]. Several lines of evidence indicate an association between lysosomal cholesterol accumulation and inflammation [9]–[12].

In order to investigate whether a macrophage-specific deletion of each scavenger receptor individually affects hepatic inflammation, *Ldlr^−/−^* mice were lethally irradiated and transplanted with bone marrow from Wild type (Wt), *Msr1^−/−^* or *Cd36^−/−^* mice and fed a high-fat-high-cholesterol (HFC) diet for 3 months. We found that CD36 and MSR1 contribute in a similar manner to the progression of NASH in the presence of high levels of plasma-modified lipoproteins. The effect of these receptors on inflammation is likely modulated via impaired cholesterol trafficking inside KCs and increased lysosomal cholesterol accumulation.

## Materials and Methods

### Ethics statement

This study was carried out in strict accordance with the recommendations in the Guide for the Care and Use of Laboratory Animals of the National Institutes of Health. The protocol was approved by the Committee for Animal Welfare of Maastricht University (Permit Number: 2006-177). The investigation conforms to the Guide for the Care and Use of Laboratory Animals published by the US National Institutes of Health (NIH Publication No. 85–23, revised 1996).

### Mice and diet

Mice were housed under standard conditions and given free access to food and water. Female *Ldlr^−/−^* mice were lethally irradiated and transplanted with Wt, *Cd36^−/−^* and *Msr^−/−^* bone marrow as previously described [4]. After a recovery period of 9 weeks, the mice were given a HFC diet for 3 months (n = 9 Wt-tp mice, n = 8 for both *Cd36* ^−/−^-tp and *Msr1* ^−/−^-tp mice), containing 21% milk butter, 0.2% cholesterol, 46% carbohydrates and 17% casein. Collection of blood and specimens, biochemical determination of lipids in plasma and liver, liver histology, RNA isolation, cDNA synthesis and qPCR, aminotransferases, oxysterols and auto-antibody titers against IgG and IgM antibodies to CuOx-LDL and MDA-LDL, as well as T15id+ IgM were determined as previously described [4].

### Electron microscopy

A detailed overview about the (post)fixation, embedding, cutting and type of electron microscope was described previously [3]. Scoring of electron microscopy (EM) pictures was performed by a trained specialist. Fifty KCs per mouse were analysed and scored. The KCs were divided into three different categories, i.e., primary lysosomes, secondary fatty lysosomes and cytoplasmic fat accumulation and the percentage of each subtype was calculated. Furthermore, the secondary fatty lysosomes were scored from 0 to 3 for their fat content, where a score of 0 indicated not positive and a score of 3 indicated extreme fat accumulation inside the lysosomes of the KCs. The same scoring index was used for the cytoplasmic fat accumulation inside KCs.

### Statistical analysis

The data were statistically analysed by performing a one-way ANOVA test with a Bonferroni post-test using GraphPad Prism for comparing Wt-tp, *Cd36^−/−^-*tp and *Msr^−/−^*-tp mice with each other. The same was done for the human samples where the different groups of patients were compared with each other. The data were expressed as mean ± SEM and considered significant at p<0.05. *, ** and *** indicate p<0.05, 0.01 and 0.001, respectively.

## Results

### Inflammation is reduced in *Cd36^−/−^-*tp and *Msr1^−/−^-*tp mice compared to Wt-tp mice

**Figure 1:**
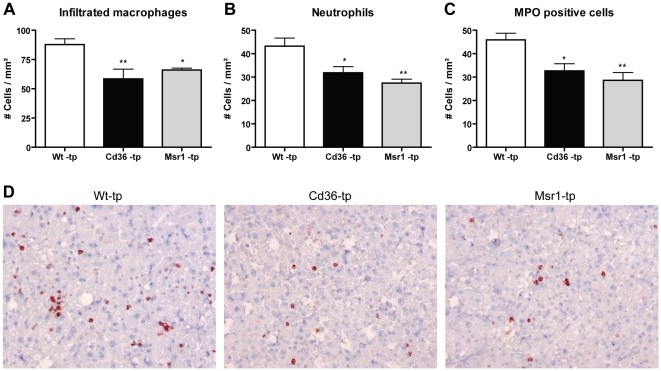
Parameters of hepatic inflammation. (A–C) Liver sections were stained for infiltrated macrophages and neutrophils (Mac-1), neutrophils (NIMP) and MPO (D) Representative pictures of Mac-1 staining (×200 magnification) after 3 months of HFC feeding in Wt-tp, *Cd36^−/−^*-tp and *Msr1^−/−^*-tp mice. *Significantly different from Wt-tp group. * and ** indicate p<0.05 and 0.01, respectively.

**Figure 2:**
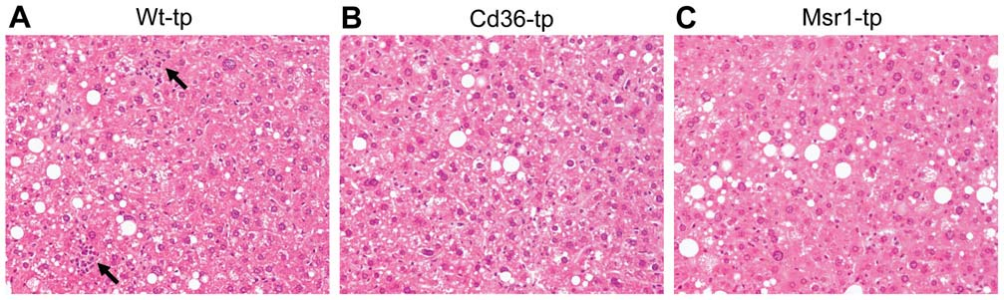
General histology of the liver. HE staining on livers of *Wt*-tp (A), *Cd36* ^−/−^-tp (B) and *Msr1* ^−/−^-tp (C) mice indicating steatosis and inflammation (arrows).

To determine whether both *Cd36* and *Msr1* are independently involved in diet-induced hepatic inflammation, *Ldlr^−/−^* mice were transplanted with *Cd36^−/−^* or *Msr1^−/−^* bone marrow and fed a HFC diet for 3 months. Immunohistochemistry revealed that infiltrated macrophages and neutrophils were significantly decreased in the livers of mice transplanted with both *Cd36^−/−^* and *Msr1^−/−^* bone marrow compared to *Wt*- transplanted (tp) mice (Fig. 1A+B+D). The same decrease was seen after staining with an antibody against myeloperoxidase (MPO), which is an oxidative stress/neutrophil marker (Fig. 1C). These data on inflammation were confirmed by a general HE staining, indicating less hepatic inflammation in *Cd36^−/−^*- and *Msr1^−/−^*-tp mice (Fig. 2).

The plasma transaminases alanine aminotransferase (ALT) and aspartate aminotransferase (AST) are considered as sensitive indicators of liver damage. Although the ALT levels were equal in all transplanted groups, the AST levels were significantly lower in *Cd36^−/−^*-tp and *Msr1^−/−^*-tp mice compared to Wt-tp mice. Moreover, the ratio of AST/ALT was lower in *Msr1^−/−^*-tp mice compared to Wt-tp mice (Table 1). In general, these data indicate that both *Cd36^−/−^*-tp and *Msr1^−/−^*-tp mice showed less liver damage.

**Table 1:** Parameters for lipid related products in liver and plasma.

	Wt-tp mice	*Cd36^−/−^*-tp mice	*Msr1* ^−/−^-tp mice
***Liver***			
Triglycerides (µg)	0.38 (±0.02)	0.40 (±0.03)	0.47 (±0.02)*
Free fatty acids (µg)	0.17 (±0.003)	0.19 (±0.006)	0.20 (±0.01)*
Cholesterol (µg)	0.08 (±0.0038)	0.10 (±0.0062)	0.11 (±0.0071)**
Lathosterol (ng/mg liver)	21.8 (±1.76)	19.71 (±2.44)	19.10 (±2.55)
Desmosterol (ng/mg liver)	16.11 (±1.06)	14.44 (±0.85)	12.62 (±0.92)*
Cholestanol (ng/mg liver)	93.03 (±8.42)	97.33 (±6.38)	126.7 (±11.22)*, #
Cholesterol esters (µg/mg liver)	14.37 (±1.58)	21.2 (±1.88)*	20.99 (±1.27)**
Free cholesterol (µg/mg liver)	8.63 (±0.28)	9.00 (±0.23)	8.16 (±0.36)
***Plasma***			
Triglycerides (mM)	1.49 (±0.16)	1.29 (±0.14)	1.27 (±0.18)
Free fatty acids (mM)	0.58 (±0.04)	0.59 (±0.06)	0.45 (±0.03)
Cholesterol (mg/dl)	1052 (±113.1)	1210 (±37.76)	1097 (±69.45)
Lathosterol (mg/dl)	0.31 (±0.03)	0.24 (±0.04)	0.17 (±0.03)**
Desmosterol (mg/dl)	0.77 (±0.06)	0.77 (±0.08)	0.52 (±0.06)*, #
Cholestanol (mg/dl)	3.03 (±0.31)	4.34 (±0.13)**	4.60 (±0.14)**
7α-OH cholesterol (ng/ml)	1.50 (±0.13)	1.02 (±0.13)*	0.92 (±0.03)**
24-OH cholesterol (ng/ml)	448.8 (±46.85)	506.5 (±28.77)	505.6 (±41.62)
27-OH cholesterol (ng/ml)	504 (±59.81)	504.8 (±42.63)	455 (±46.12)
MDA-LDL IgM (RLU)	86348 (±4857)	89405 (±6189)	113896 (±2889)***,##
CuOx-LDL IgM (RLU)	31987 (±6331)	62903 (±11844)*	102371 (±6341)***,##
T15id+ (IgM) (RLU)	3726 (±395.2)	9000 (±1815)*	19079 (±3412)**, #
ALT (U/l)	61 (±9.35)	58.43 (±3.40)	65.5 (±3.53)
AST (U/l)	129.5 (±13.77)	79 (±14.3)*	84 (±6.52)*
AST/ALT	2.25 (±0.38)	1.52 (±0.15)	1.28 (±0.07)*

* Significantly different from *Wt*-tp group. *, ** and *** indicate p<0.05, 0.01 and 0.001, respectively. RLU: Relative light unit.

# Significantly difference between *Cd36^−/−^*-tp and *Msr1^−/−^*-tp mice. # and ## indicate p<0.05 and 0.01 respectively.

### The reduced inflammatory response in *Cd36^−/−^*-tp and *Msr1^−/−^*-tp mice is not correlated with the changes in lipid levels

To determine whether deletion of *Cd36* and *Msr1* on bone marrow cells affected steatosis, liver lipid levels were analysed in order to investigate the extent of steatosis present in these mice. Table 1 show that liver triglycerides (TG), free fatty acids (FFA) and total cholesterol were all higher in the *Msr1* ^−/−^-tp mice compared to *Wt*-tp mice. The amount of cholesterol esters (CE) in the liver was elevated in both *Cd36^−/−^*-tp and *Msr1^−/−^*-tp mice compared to *Wt*-tp mice. Desmosterol, a cholesterol synthesis marker, was decreased in the *Msr1* ^−/−^-tp mice compared to Wt-tp mice, whereas the cholesterol absorption marker cholestanol was higher in *Msr1* ^−/−^-tp mice compared to *Wt*-tp and *Cd36^−/−^*-tp mice (Table 1). Nevertheless, HE staining (Fig. 2) and Oil red O staining (data not shown) revealed no differences between any of the groups. Thus, the observed differences found in hepatic steatosis between *Wt*-tp, *Cd36^−/−^*-tp and *Msr1^−/−^*-tp mice were mild and not consistent.

Table 1 shows that plasma TG and total cholesterol were similar in all transplanted groups. Plasma FFA levels were significantly lower in *Msr1^−/−^*-tp mice compared to Wt-tp mice. Although the total amount of cholesterol did not differ between the groups, the cholesterol synthesis markers lathosterol and desmosterol were significantly lower in the plasma of *Msr1^−/−^*-tp mice compared to Wt-tp mice. Moreover, the cholesterol absorption marker cholestanol was significantly higher in both *Cd36^−/−^*-tp and *Msr1^−/−^*-tp mice compared to Wt-tp mice. Plasma levels of 7α-hydroxy (OH) cholesterol were decreased in both *Cd36^−/−^*-tp and *Msr1^−/−^*-tp mice compared to *Wt*-tp mice, while the other oxysterols (24-OH and 27-OH cholesterol) were not different from *Wt-*tp mice. Overall, these plasma lipid data were inconclusive and did not reveal any major differences between *Wt*-tp, *Cd36^−/−^*-tp and *Msr1^−/−^*-tp mice.

In order to investigate whether natural antibodies, known to decrease atherogenesis, are affected by deletion of *Cd36* and *Msr1*, the protective immunoglobulin M auto-antibody levels against MDA-LDL, Cu-OxLDL and T15id+ were measured and showed significantly higher levels in *Msr1^−/−^*-tp mice compared to Wt-tp mice and *Cd36^−/−^*-tp mice. The Cu-OxLDL and T15id+ IgM auto-antibodies were also elevated in *Cd36^−/−^*-tp mice compared to Wt-tp mice. Thus, both *Cd36^−/−^*-tp and *Msr1^−/−^*-tp mice have an increased protective phenotype compared to Wt-tp mice.

### No difference in the foamy appearance of Kupffer cells

**Figure 3:**
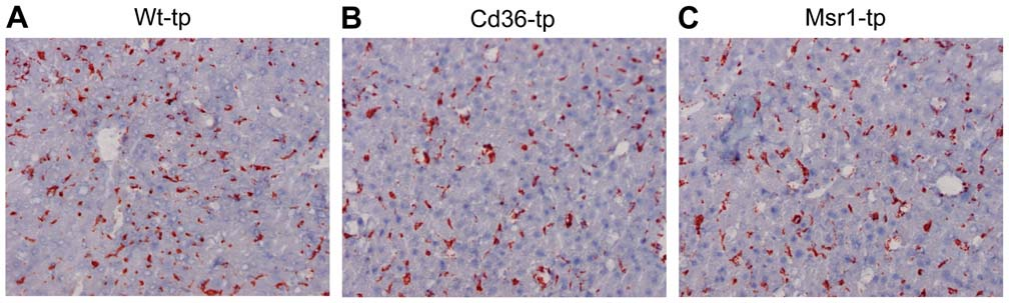
Foamy Kupffer cells. CD68 staining of Kupffer cells in liver of *Wt*-tp (A), *Cd36* ^−/−^-tp (B) and *Msr1* ^−/−^-tp (C) mice.

To characterize the KCs, immunohistochemistry for CD68 (macrophage marker) was performed. This staining revealed no differences in the size or presence of KCs between Wt-tp, *Cd36^−/−^*-tp and *Msr1^−/−^-*tp mice (Fig. 3A–C). Moreover, CD68 mRNA expression showed similar results (data not shown).

### *Cd36* ^−/−^-tp and *Msr1* ^−/−^-tp mice have decreased lysosomal cholesterol storage inside the KCs compared to Wt-tp mice

**Figure 4:**
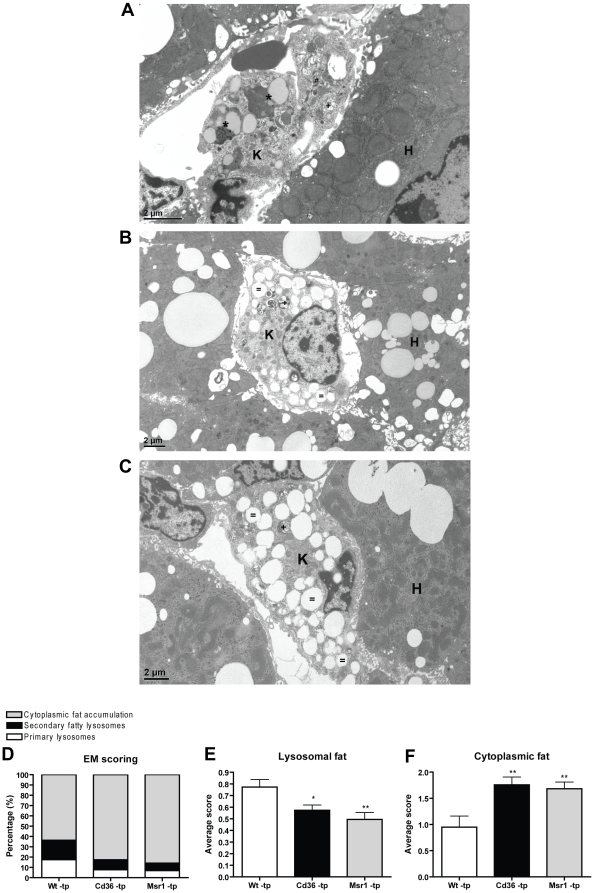
Electron microscopic views of Kupffer cells (K) after 3 months of a high fat diet. (A) The Kupffer cell of Wt-tp mice shows several fatty lysosomes (*). Primary lysosomes (#) and secondary lysosomes without fat accumulation (+) can be seen as well. Kupffer cells of *Cd36* ^−/−^ -tp (B) and *Msr1* ^−/−^ -tp (C) mice show massive cytoplasmic lipid accumulation ( = ). H: hepatocytes. (D) Percentage of Kupffer cells with primary lysosomes, secondary fatty lysosomes and cytoplasmic fat accumulation. (E+F) Arbitrary scoring of secondary fatty lysosomes and cytoplasmic fat accumulation inside Kupffer cells. *Significantly different from Wt-tp group. * and ** indicate p<0.05 and 0.01, respectively.

To determine whether the reduced inflammatory response in *Cd36^−/−^*-tp and *Msr1^−/−^*-tp mice was associated with decreased lysosomal fat accumulation, detailed analyses of the KCs were performed using electron microscopy (EM) (Fig. 4A–C). Interestingly, lipid distribution inside these cells revealed a clear difference between Wt-tp and *Cd36* ^−/−^ -tp/*Msr1* ^−/−^ -tp mice (Fig. 4D). *Cd36* ^−/−^-tp and *Msr1* ^−/−^-tp mice showed decreased lysosomal cholesterol storage inside the KCs compared to Wt-tp mice, whereas the first two had increased cytoplasmic fat storage compared to Wt-tp mice (Fig. 4E+F). Moreover, Wt-tp mice had more primary and secondary lysosomes compared to *Cd36* ^−/−^-tp and *Msr1* ^−/−^-tp mice.

## Discussion

Our study showed for the first time that the uptake of modified lipids *in vivo* leads to lysosomal cholesterol accumulation in KCs. By specifically inactivating one of the two predominant scavenger receptors on macrophages, we established that the contribution of each one of these two receptors to hepatic inflammation is similar. Internalization of lipids by these receptors leads to increased levels of cholesterol in lysosomes and increased hepatic inflammation.

### CD36 and MSR1 contribute in a similar manner to the progression of NASH

Currently, the risk factors that drive hepatic inflammation during the progression of NASH are largely unknown. We previously showed that hyperlipidemic mice are more sensitive to developing early diet-induced NASH [3]. We also demonstrated that the increased sensitivity of these mice towards developing NASH is related to the expression of *Msr1* and *Cd36* [4].

Scavenger receptors MSR1 and CD36 have been identified as the principal receptors responsible for the uptake of modified lipids and cholesterol into macrophages [13]. MSR1 was found to account for the majority (80%) of the macrophage uptake of acetylated low density lipoproteins (acLDL), but it also binds oxidized LDL (oxLDL), albeit with lower affinity. CD36 binds oxLDL rather than acLDL, and it is involved in oxLDL-induced JNK activation. While the relative affinities of MSR1 and CD36 to modified and oxidized lipids are different, the effects on inflammation in our study were similar. These results are surprising, since it was found that cultured macrophages incubated with oxLDL showed lysosomal cholesterol accumulation while incubation with acLDL did not lead to lysosomal cholesterol accumulation but rather to cytoplasmic cholesterol storage inside macrophages [8]. Thus, it is possible that in contrast to the *in vitro* data, the affinity of both receptors towards oxLDL *in vivo* is similar. Another possible explanation is that the markedly increased levels of oxLDL in the plasma of *Ldlr^−/−^* mice fed the HFC diet exceeded the maximal amount of cholesterol that can be taken up by KCs and trapped inside their lysosomes. The scavenger receptors CD36 and MSR1 differ from each other not only in their relative affinities to modified and oxidized lipids, but also in their signalling pathways and other important functions triggered by the binding and uptake of modified LDL, such as innate immune responses, cellular adhesion, and the phagocytosis of apoptotic cells [7]. These functions may also be involved in the development of NASH. The similarity in the degree of inflammation between the two models suggests that this inflammation is a consequence of the two receptors sharing similar modified lipid internalization mechanisms, rather than each receptor having its own specialized functions. In line with this hypothesis, previous studies with MSR1 and CD36-knockout mice showed that disruption of each receptor pathway partially inhibited the uptake of modified LDL in macrophages and retarded atherosclerosis progression in a similar manner [14]–[16]. All together, this study provides evidence of a central contribution of both of these receptors to the pathogenesis of NASH. Further investigations are necessary to determine the exact interaction between CD36 and MSR1 upon the internalization of lipids.

### Hepatic inflammation is correlated with increased lysosomal cholesterol accumulation

Interestingly, it was shown that lysosomal cholesterol accumulation *in vitro* was associated with increased pH inside lysosomes [17], which can lead to partial lysosomal enzyme inactivation and an inflammatory response. Similarly, it was shown that in foam cells of advanced atherosclerotic plaques, cholesterol is not transferred into the cytoplasm but rather accumulates in the lysosomes of the macrophages [18]. Several lines of evidence further indicate an association between lysosomal cholesterol accumulation and inflammation: first, lysosomal acid lipase (LAL) is the essential enzyme for the hydrolysis of triglycerides and cholesteryl esters in lysosomes. Similar to our observations in hyperlipidemic mice, a deficiency of this enzyme was found to lead to lysosomal cholesterol accumulation and inflammation [12]. Moreover, exogenous LAL administration in *Ldlr^−/−^* mice led to a significant reduction in hepatic inflammation and lipid accumulation. Furthermore, the macrophage-specific expression of human LAL was found to correct inflammation in *Lal^−/−^* mice [12]. Likewise, patients with mutations in Niemann-Pick type C (NPC) 1 and 2, proteins which facilitate the movement of cholesterol from the lysosomes to the cytoplasmic compartment, commonly have a very poorly functioning liver, and about 10% of these patients dies from liver failure [19], [20]. Second, lysosomes have been assigned a central role in many processes involving tissue injury and inflammation [21]. Likewise, increased lysosomal cholesterol accumulation contributes to disturbed autophagy, a process that has recently emerged as an important regulatory pathway of the innate immune response [22]. Finally, it was recently demonstrated that oxLDL has the potential to damage lysosomal membranes and thereby prime cells for inflammation [23].

Taken altogether, our novel observations point towards the role of scavenger receptors during hepatic inflammation, resulting in abnormal cholesterol trafficking in KCs. These data provide a new basis for prevention and treatment of NASH.
